# The European Portuguese version of the Reproductive Concerns After Cancer Scale for male cancer survivors: A preliminary psychometric validation study

**DOI:** 10.1192/j.eurpsy.2024.657

**Published:** 2024-08-27

**Authors:** J. Silva, A. Bártolo, I. S. Silva, A. F. Oliveira, A. Louro, A. Torres, I. M. Santos, S. Monteiro

**Affiliations:** ^1^University of Aveiro, Department of Education and Psychology, Aveiro; ^2^Portucalense University, I2P—Portucalense Institute for Psychology, Porto; ^3^Piaget Institute, RECI - Research Unit in Education and Community Intervention, Viseu; ^4^TERRA Associate Laboratory, University of Coimbra, Centre for Functional Ecology-Science for People & the Planet (CFE), Coimbra; ^5^Department of Education and Psychology, University of Aveiro, CINTESIS@RISE, Aveiro; ^6^Department of Psychology and Education, Faculty of Social and Human Sciences, University of Beira Interior (UBI), Covilhã; ^7^Department of Education and Psychology, University of Aveiro, William James Center for Research, Aveiro; ^8^Department of Social and Management Sciences; ^9^Center for Global Studies, Open University, Lisbon, Portugal

## Abstract

**Introduction:**

Cancer treatments can affect male fertility. However, the reproductive concerns of this population remain little explored. There is a need to invest in understanding how concerns related to fertility and parenting affect psychosocial adjustment, in order to improve counseling in this context. To this end, it is a priority to provide reliable and valid measures for assessing this construct.

**Objectives:**

This study aimed to translate, adapt and preliminarily explore the psychometric properties of the Portuguese version of the Reproductive Concerns After Cancer Scale - Male Version (RCAC-M).

**Methods:**

Translation and back-translation were carried out by two independent translators. A reconciled version was obtained and evaluated by a panel of experts who ensured its cultural adaptation. Before studying the psychometric properties, a pre-test was carried out involving a focus group of 5 male cancer survivors who assessed the adequacy of the measure. The preliminary validation included 32 male cancer survivors aged between 18 and 55. Recruitment was carried out by providing an online questionnaire. A principal component analysis was carried out to explore the factor structure of the measure and to analyze the reliability and convergent validity of the measure.

**Results:**

The results showed good internal consistency of a version consisting of 17 items, grouped into four factors: fertility potential, child health and future life, personal health and future life, and acceptance. Significant moderate associations were found between reported concerns and other constructs that are consistently related to this variable in the literature, namely the importance of parenting and symptoms of anxiety and depression.

**Conclusions:**

The original structure of the scale was not corroborated. However, this study suggests the promising character of the Portuguese version of the RCAC-M as a reliable and valid tool for assessing the reproductive concerns of male cancer survivors.

**Disclosure of Interest:**

None Declared

